# Blastocyst contractions are strongly related with aneuploidy, lower implantation rates, and slow-cleaving embryos: a time lapse study

**DOI:** 10.5935/1518-0557.20190053

**Published:** 2020

**Authors:** Eduardo Gazzo, Fernando Peña, Federico Valdéz, Arturo Chung, Marcelo Velit, Mario Ascenzo, Ernesto Escudero

**Affiliations:** 1 INMATER Fertility Clinic, Lima, Peru; 2 Genomics Perú, Lima, Peru

**Keywords:** time-lapse monitoring, blastocyst contraction, solo expanding, single-embryo transfer

## Abstract

**Objectives:**

This study aimed to identify human blastocyst contraction patterns and their correlations with ploidy status (PGT-A analysis), the time it took for embryos to reach the blastocyst stage, and pregnancy rates.

**Methods:**

The study included 912 embryos from 270 patients seen in our center. All embryos were cultivated in an Embryoscope incubator. An NGS platform was used to test 778 of the 912 embryos initially included in the study for aneuploidy at a reference laboratory. Blastocyst contractions were evaluated using the embryo drawing tool to compute percent contraction. A total of 182 single-embryo transfers were performed. The mean age of the included patients was 30.44 years (24-39 years).

**Results:**

The embryos were divided into two groups, the first with embryos that contracted (CT group) and the second with embryos that did not contract, herein referred to as expanding-only embryos or solo expanding (SE group). In terms of ploidy status, 58.33% of the embryos in the SE group were euploid, while 53.58% of embryos in the CT group were aneuploid. The difference between the groups was statistically significant (*p*=0.029), showing that embryos that do not contract have a higher chance of being euploid than embryos that contract. Pregnancy rates were also significantly higher among embryos in the SE group than in the CT group (63.10% *vs.* 46.67%; *p*=0.012). Finally, we saw that embryos in the CT group took significantly longer to reach the blastocyst stage compared to embryos in the SE group (*p*=0.004). Patient age was not significantly different between the CT and SE groups, indicating that age might not be a factor in embryo contraction.

**Conclusion:**

Two of the traits for which the embryos included in this study were compared were statistically different. Embryos in the CT group had lower implantation rates, took longer to reach the blastocyst stage, and had a higher chance of being aneuploid, regardless of maternal age. Therefore, embryo contraction might be a useful parameter in the selection of embryos for transfer.

## INTRODUCTION

One of the most significant challenges in IVF revolves around selecting embryos to produce single pregnancies and healthy babies within the shortest time possible. This is why IVF labs are constantly searching for new tools to allow them to achieve this objective.

One of the tools developed to achieve this goal is time-lapse technology. Over the past few years, time-lapse has been increasingly used to improve the outcomes of embryo selection from traditional morphological assessment. Time-lapse technology allows embryologists to follow the events of embryo development in ways never seen before (Meseguer *et al*., 2012; Rubio *et al*., 2014; VerMilyea *et al*., 2014).

One of the events observable with time-lapse technology is embryo contraction. First reported in 1929 (Niimura, 2003), embryo contraction is defined as a spontaneous separation of the pellucid zone and the trophectoderm that occurs only from the early blastocyst stage forward (Marcos *et al*., 2015). The reason for embryo contraction remains unknown.

Embryo expansion is caused by an inflow of extracellular liquid through aquaporin water channels (AQP3/AQP9) into trophectoderm cells due to increases in ion concentrations in the blastocoel caused by the sodium/potassium pump (Watson *et al*., 2004). On the other hand, contractions result from fluid leaving through weak tight junctions (Marcos *et al*., 2015).

Embryo contraction and its effects on embryo development and viability has been the topic of a handful of studies. The literature indicates that embryo contraction may inhibit blastocyst hatching in mice (Niimura, 2003), while in humans it may be associated with lower implantation and slower division rates (Marcos *et al.,* 2015; Bodri *et al.,* 2016; Niimura, 2003).

No morphological parameter can fully predict the ploidy status of an embryo, leaving to molecular biology the role of finding euploid embryos for transfer. Although several morphological traits may be associated with higher aneuploidy rates (Basile *et al.,* 2014), they have little predictive value in embryo selection when used alone (Rienzi *et al*., 2015). However, when PGT-A and time-lapse technology are combined, there is a clear improvement in implantation rates (Yang *et al*., 2014).

This study aimed to evaluate embryo contractions in embryos categorized as normal after PGT-A and to assess whether there is a relationship between embryo contraction, aneuploidy, and implantation rate.

## MATERIALS AND METHODS

### Patients and embryos

The study included 912 embryos from 270 patients seen in our IVF center. The mean age of the included patients was 30.44 years (24-39 years). PGT-A was performed on 778 embryos that reached the blastocyst stage categorized as good quality embryos fit for biopsy.

### Ovarian Stimulation

Ovarian stimulation was administered with 150 to 300 IU/day of FSH and/or HMG during the first two to five days of the cycle depending on patient age, BMI, and previous response to stimulation (when known). Doses were administered and adjusted after ultrasound evaluation every 2-3 days. Patients also received GnRh antagonists and stimulation continued until the main follicles reached 18mm in diameter. Then patients were administered hCG and/or a GnRH agonist and 36 hours later follicular aspiration was performed.

### Oocyte retrieval and in vitro fertilization

Ultrasound-guided oocyte retrieval was performed 36 hours after the trigger with the patients under general anesthesia. A 17G needle (Ops Classic avec Robinet, Laboratoire CCD) was used in the procedure.

Retrieved oocytes were washed with *Global^®^ total with HEPES* (LifeGlobal, Canada) medium and cultured in *Global^®^ total for Fertilization* (LifeGlobal, Canada) medium at concentrations of 5.6% CO_2_ and 5.0% O_2_ at 37ºC. All samples were incubated in *K-Systems^®^ invi cell G210* incubators for about three hours before oocyte mechanic denudation. Denudation was performed with a glass pipette with hyaluronidase and oocytes were washed with *Global^®^ total with HEPES* (LifeGlobal, Canada) medium. Mature oocytes were cultured for 40 minutes before microinjection.

### Embryo culture

Evaluation was performed 18-20 hours after insemination. All fertilized oocytes were first transferred to an Embryoslide^®^ (Vitrolife, Denmark) dish equilibrated the night before, and then placed in the *Embryoscope^®^* (Vitrolife, Denmark). Each Embryoslide^®^ (Vitrolife, Denmark) dish has 12 incubation wells, each containing 20 µL of GTL medium (Vitrolife, Canada) covered with 1.8ml of mineral oil *OVOIL* (Vitrolife, Canada) to avoid evaporation of the medium. The embryos were monitored for five or six days and were only removed from the incubator on day 4, when the embryos set for PGT-A analysis underwent assisted hatching. Only embryos that reached the expanded blastocyst or hatching stage were biopsied.

### Assisted hatching

All embryos scheduled for PGT-A evaluation underwent assisted hatching on day 4 of culture.

### Time-lapse notes and video review

The incubator image acquisition system was pre-programmed to take pictures every 10 minutes, with a resolution of 1000 x 1000 pixels in seven focal planes distant 15 µm between each other, to ensure appropriate embryo morphology evaluation at the time of video analysis.

Immediately after the embryos were taken from the *EmbryoScope*^®^, two expert embryologists (E.G. and F.P.) reviewed the videos using the *EmbryoViewer^®^* software.

Blastulation start time and time of biopsy were recorded for all embryos. Blastulation start time was defined as the moment in which the pellucid zone of the embryo began to thin and the blastocoel began to show signs of cavity formation. All embryos that reached the blastocyst stage were analyzed and graded based on the criteria developed by Gardner & Schoolcraft (1999). Embryo parameters were entered into the EmbryoScope software to inform the choice of embryos sent for vitrification (Criotech Co, Ltd) and biopsy to study the trophectoderm.

### Definition of the measurement of contractions

We used the Embryoviewer drawing tool to assess embryo contractions based on a modification of the method described by Marcos *et al*. (2015) that allowed the categorization of contractions into different groups.

**Figure 1:**
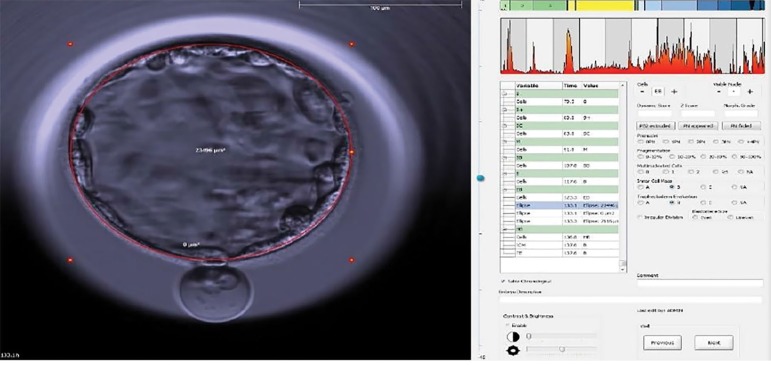
Time of first measurement: expanded blastocyst before contraction. Event time: h: 133.1 Measurement of the red ellipse = 13.737 µM.

**Figure 2:**
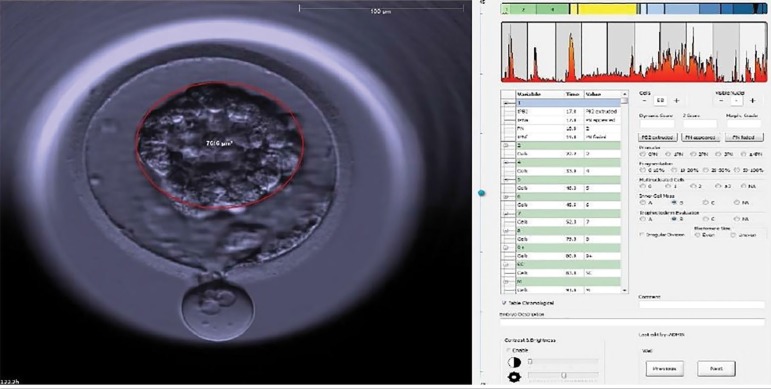
Time of second measurement: the same blastocyst at the point of greatest contraction. Event time: h: 133.2 Measurement of the red circle = 4.754 µM. This blastocyst contracted by 65.39%.

When performing the morphokinetic evaluation of the embryo, special attention was paid from the early blastocyst stage forward. When a contraction occurred, the video was paused so that the intensity of the contraction was quantitatively determined with the aid of the "draw ellipse" tool. Contraction was calculated as the area of the blastocyst immediately before contraction minus the area of the contracted blastocyst. Percent contraction was calculated as the number resulting from the difference between the two areas divided by the greater area of the two. The calculation was performed only once taking the largest contraction into account when blastocysts had more than one contraction. Figures 1 and 2 show the percent contractions of all embryos that presented contractions.

### Blastocyst biopsy

Embryo biopsy was performed immediately after the embryos were taken from the Embryoscope^®^ using an OLYMPUS IX73 inverted microscope, a LIKOS (Hamilton Thorne) laser, TransferMan 4r (Eppendorf) micromanipulators, and *HOLDING MPH-MED-30* (Origio) and *BIOPSY MBB-FP-M-30* (Origio) micropipettes. Laser power was set on *Validation* mode (100% *power* - pulses of 430 microseconds) and no more than four laser shots were used to separate trophectoderm cells. After biopsy, *tubing* was performed according to the recommended protocol from the genetics laboratory (*Genomics Perú)*. The embryos were kept vitrified until the results from PGT-A analysis were available.

### Vitrification and thawing

Biopsied embryos were vitrified following the cryotech method and stored in liquid N_2_ until transfer.

Euploid embryos were selected and thawed using the cryotech protocol and cultured between 60 and 120 minutes in Embryoglue media (Vitrolife*,* Canada) before transfer.

### Preimplantation genetic testing

Of the 912 embryos evaluated with the Embryoscope^®^, 778 were biopsied for PGT-A. Preimplantation genetic testing for aneuploidy was performed by means of next generation sequencing (NGS) in a Miseq^®^ (Illumina^®^ Inc) sequencer. Complete genome amplification was performed using the Sureplex method, following manufacturer instructions. Illumina^®^ Veriseq kits were used for library preparation and molecular cytogenetic data analyses were done using the Illumina BlueFuse^®^ software. Our partner genetics laboratory, Genomics Perú, performed all genetic tests and ensuing data analyses.

### Statistical analysis

The chi-square test was used in the analysis of nonparametric proportions. Parameters following a normal distribution were treated using the analysis of variance (ANOVA) test. Results were considered statistically significant when *p*<0.05. Software *Statistical Package for the Social Sciences* 24.0 (SPSS Inc.) was used in data analysis and interpretation.

## RESULTS

**Graph 1:**
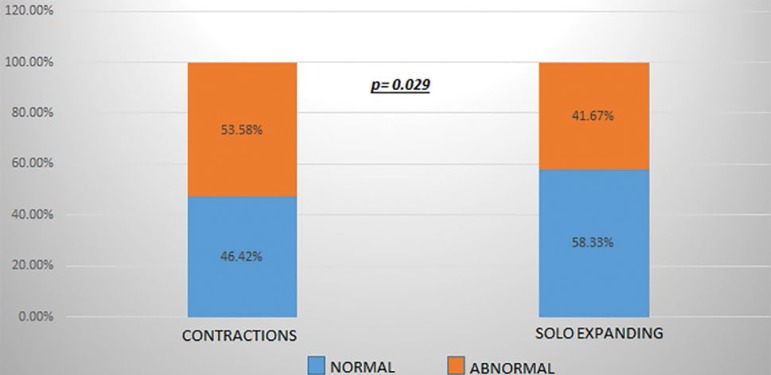
Embryo euploidy rate according to type of movement: contraction vs. expansion only (“solo expanding”). Euploidy rates were significantly different between the SE and CT groups (*p*=0.029).

A total of 257 of the 912 assessed embryos had contractions (28.18%). Aneuploidy tests were performed in 196 of the 257 embryos. PGT-A results revealed that 91 (46.42%) embryos were euploid. Nearly three fifths (58.33%) of the 534 embryos in the SE group screened with PGT-A were euploid (Graph 1).

**Graph 2:**
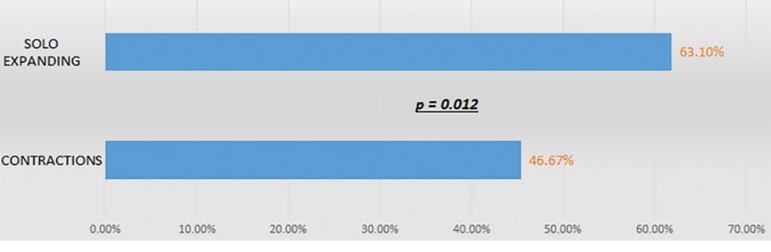
Ongoing pregnancy in euploid embryos according to type of movement: contraction vs. expansion only (“solo expanding”).

Eighty-four euploid embryos in the SE group and 30 euploid embryos in the CT group were transferred. Fifty-six of the 84 embryo transfers in the SE group resulted in patients with positive ß-hCG tests and 53 (63.10%) led to ongoing pregnancies. Ongoing pregnancies were recorded in 46.67% of the 30 embryo transfers in the CT group. The proportions of ongoing pregnancy seen in the CT and SE groups were significantly different (*p*=0.012) (Graph 2).

**Graph 3:**
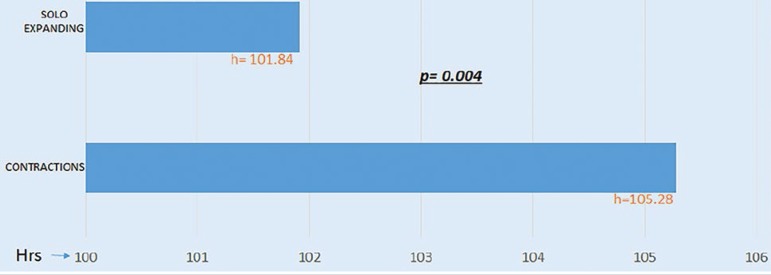
Time to blastocyst in euploid embryos (h) according to type of movement: contraction *vs*. expansion only (“solo expanding”).

We also looked into the time the embryos took to reach the initial stages of blastulation and found that the 81 euploid embryos in the CT group took on average 105.28 hours to develop a pellucid zone. In the SE group, 296 euploid embryos took on average 101.84 hours to develop a pellucid zone, revealing a statistically significant difference between the groups (*p*= 0.004) (Graph 3).

**Graph 4:**
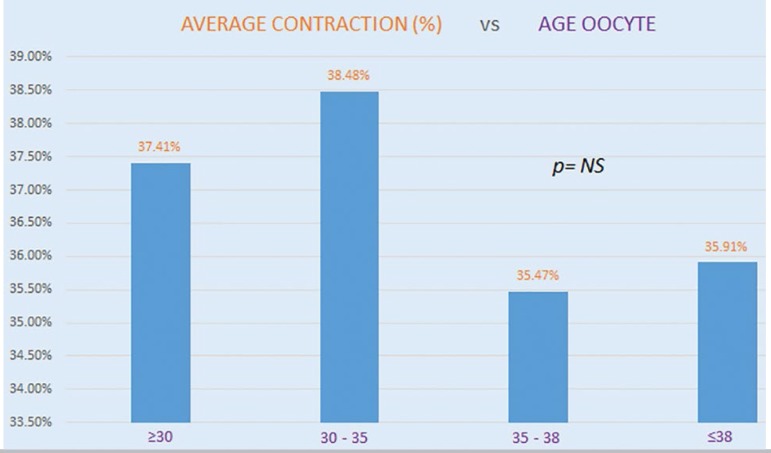
Oocyte age and presence of contraction were not statistically correlated. Embryos showing contraction were grouped based on oocyte age. Mean embryo contraction is shown for each group.

When we looked at the age of the oocytes used in this study, we did not find significant correlations between age and embryo contractions. This indicated that maternal age and embryo contractions might not be correlated. Other findings suggest that embryo contractions might be associated with genetic causes (Graph 4).

**Graph 5:**
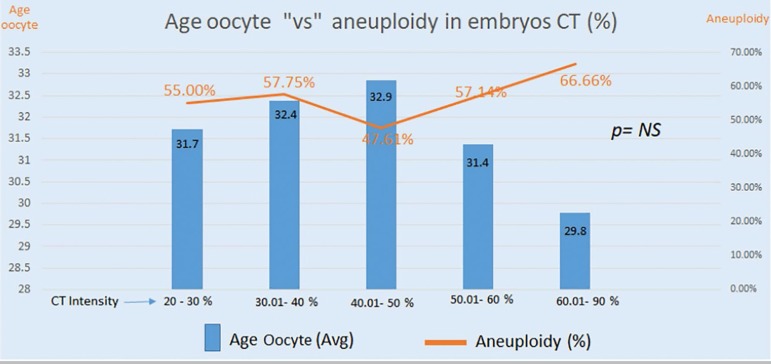
Embryos divided into five groups based on percent contraction (blue bars). Group 1 (n=55); Group 2 (n=59); Group 3 (n=42); Group 4 (n=14); Group 5 (n=9). The discrepancies seen in Groups 4 and 5 may be ascribed to the small number of embryos in each of the two groups.

Graph 5 shows embryos that had contractions divided into five groups based on percent contraction, as follows: Group 1= 20-30%; Group 2= 30-40%; Group 3= 40-50%; Group 4= 50-60%; and Group 5= 60-90% contraction. The median age of patients and proportions of aneuploid embryos (orange line) were not significantly different.

## DISCUSSION

The primary goal of this study was to assess embryo contraction as a possible marker of implantation with the aid of an Embryoscope incubator and PGT-A testing. Niimura (2003) observed the occurrence of weak contractions in murine embryos often close to embryo hatching, and saw that embryos with higher percent contraction had a greater chance of presenting problems during hatching. The authors found that low percent contraction (less than 20%) played an important role during embryo hatching, while higher percent contraction (greater than 20%) had an adverse effect on hatching. Our data indicated that this finding does not apply to human embryos, since we found no significant correlation between lower/higher percent contraction and embryo hatching.

Marcos *et al*. (2015) investigated embryo contractions and separated subjects into two groups - Collapsed embryos (more than 50%) and Contraction embryos (less than 50%). The authors saw that embryos that collapsed had lower implantation rates than embryos that only contracted. Differently from Marcos *et al*. (2015), we measured the difference in the area of the embryo after contraction to calculate percent contraction. This allowed us to evaluate embryo contraction regardless of the shape of the embryo or contraction.

Bodri *et al*. (2016) conducted a similar investigation and reported similar and dissimilar findings. The first difference was the proportion of embryos that presented contraction: Marcos *et al.* (2015) reported that 19% of the embryos contracted, while Bodri *et al*. (2016) reported a proportion of 46%. In our study, 28.18% of the embryos contracted. Another difference was that Bodri *et al*. (2016) reported lower quality among embryos with contractions, while Marcos *et al.* (2015) reported no difference in quality. The main result shared by Bodri *et al*. (2016), Marcos *et al.* (2015), and our study was that embryos showing contractions always yielded lower pregnancy rates.

Our study proposed a different method to look into embryo contraction vis-à-vis previous publications. First, we considered two morphokinetic patterns mentioned earlier: embryo contraction (when the trophectoderm separates form the pellucid zone and expanding-only embryos (when the trophectoderm does not separate from the pellucid zone). We used the area of contraction so as to increase the accuracy of the measurements in relation to previous methods and report percent contractions. Percent contraction varied significantly among embryos with contractions (8.59%-89.95%). This allowed us to categorize embryos in the CT group according to percent contraction and study whether the intensity of contraction had been impacted by maternal age or chromosomal configuration.

This novel way of evaluating embryo contraction revealed that maternal age and embryo contraction were not associated in any of the five groups studied. We also saw that the pregnancy rates of euploid embryos in the CT (46.67%) and SE (63.10%) groups were significantly different in favor of the embryos in the SE group. This finding supports what the study by Meseguer *et al*. (2011) showed, in which lower pregnancy rates were observed on embryos showing contraction compared with expanding-only embryos.

Embryos showing contraction took significantly longer to reach the blastocyst stage (105.28h *vs.* 101.84h), spent more time in cleavage, and had slower division rates. Contraction extended the time for which embryos in the blastocyst stage had to be kept in the incubator and delayed PGT-A testing. This finding led us to think that embryo contraction might be dictated by genetic factors, early embryo development, or even ploidy status.

Our results confirmed what Marcos *et al*. (2015) described about embryos that showed contraction, supporting that these embryos developed at a much slower rate than their counterparts that only expanded. Our findings also supported the recommendation described by Marcos *et al*. (2015) of selecting only embryos that did not contract during their development to improve implantation rates. The high demand for energy and cellular stress connected with embryo contraction may potentially affect implantation rates. More research is needed on the subject.

A total of 778 of 912 embryos included in our study underwent assisted hatching on day 4 of development. Contraction was observed in 27.12% of these embryos. For purposes of comparison, contraction was seen in 34.33% of the embryos that did not undergo assisted hatching. Unfortunately, the number of embryos used in the comparison between the two groups was too small to allow definitive conclusions to be drawn, although it indicated that embryos submitted to assisted hatching might present lower contraction rates.

We believe that contraction should be taken into consideration in the selection of embryos for transfer. As our study showed, embryos that presented contraction had lower implantation and higher aneuploidy rates when compared to embryos that only expanded. Despite our results, contraction cannot predict the ploidy status of an embryo or replace PGT-A as a tool in embryo selection.

It is the conclusion of our team that more prospective randomized studies are needed to further evaluate the impact of assisted hatching in the process of embryo contraction and possibly support the findings reported in this study.
